# Metastatic Testicular Seminoma in a Patient With Down Syndrome Presenting As Extensive Deep Venous Thrombosis

**DOI:** 10.7759/cureus.29037

**Published:** 2022-09-11

**Authors:** Meredith Hengy, Amita Hinge, James P Purtell, Kathren Shango, Joshua Collins

**Affiliations:** 1 Internal Medicine, Wayne State University School of Medicine, Detroit, USA; 2 Internal Medicine, Henry Ford Health System, Detroit, USA

**Keywords:** cancer screening, testicular cancer, down syndrome, deep vein thrombosis, seminoma

## Abstract

Testicular cancer, particularly seminoma, is associated with Down syndrome. In cognitively impaired patients, the typical presenting signs of testicular cancer may be missed, and atypical presenting features may be the only clue to the diagnosis. In this report, we present the case of a 38-year-old male who presented with extensive deep vein thrombosis in the setting of seminoma.

## Introduction

Testicular cancer has been found to be associated with Down syndrome (DS) with seminoma being the most common type [1,2]. A painless testicular mass is the most common first presenting sign in otherwise healthy patients. Atypical presenting symptoms of testicular cancer such as unexplained deep vein thrombosis (DVT) may occur in patients with cognitive impairment or those in a non-verbal state; these symptoms may be the only clue to diagnosis [3]. Failure of patients to express early symptoms may lead to delayed diagnosis and poor outcomes. We present the case of a 38-year-old male patient with DS presenting with extensive DVT in the setting of seminoma. 

## Case presentation

A 38-year-old male with a past medical history significant for DS presented to the emergency department with generalized weakness and left lower extremity swelling for five days. The patient was non-verbal at baseline, secondary to his history of DS, and he was represented by a legal guardian. The guardian reported that the patient had difficulty with walking and had refused to get out of bed for five days. The patient arrived hemodynamically stable and afebrile. 

**Figure 1:**
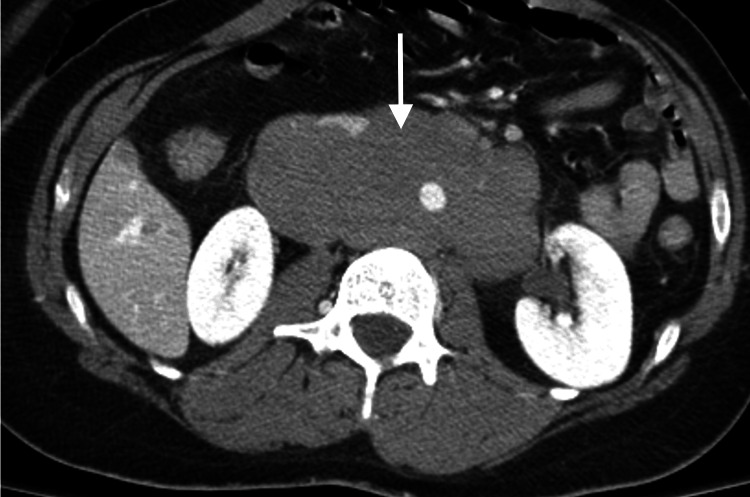
CT scan of the abdomen with contrast demonstrating extensive DVTs in the left hemipelvis (white arrow) CT: computed tomography; DVT: deep vein thrombosis

On physical examination, the patient was found to have significant left lower extremity edema and erythema when compared to the right. Venous doppler ultrasound (US) was obtained and showed totally occluding acute DVT of the left upper and lower extremities as well as totally occluding acute superficial venous thrombosis of the left great saphenous vein. Vascular surgery was consulted and determined the patient to not be a candidate for acute intervention. Computed tomography (CT) of the abdomen and pelvis with contrast demonstrated findings of extensive DVTs in the left hemipelvis, significant lymphadenopathy throughout the abdominal cavity, and new hepatic lesions, concerning for metastatic disease (Figure 1). Chest x-ray demonstrated a new focal mass at the right supra-hilar mediastinum. CT of the head was negative for metastasis. 

**Figure 2:**
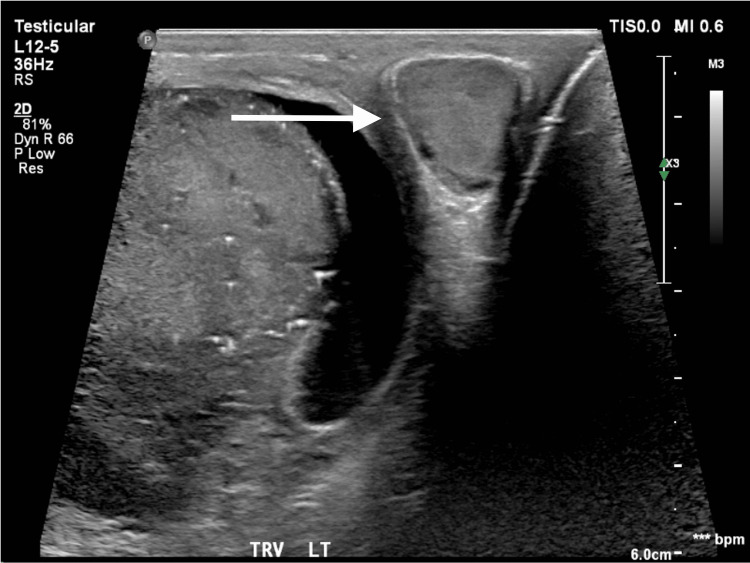
Ultrasound of the scrotum demonstrating a homogenous mass

Physical examination was repeated in search of the primary malignancy. Testicular exam was significant for a right testicular mass and scrotal swelling. US of the scrotum revealed an enlarged, heterogenous and vascular right testis concerning for neoplasm (Figure 2). Biopsy of a retroperitoneal lymph node revealed unspecified, high-grade malignant neoplasm. Tumor markers included elevated beta HCG of 130 mIU/mL, lactate dehydrogenase (LDH) of 278 U/L, and normal alpha-fetoprotein. Radical right-sided orchiectomy was performed and a biopsy revealed a 9 cm pure seminoma with invasion into the spermatic cord, determined to be stage IIIC. The patient received four cycles of cisplatin and etoposide with a slight decrease in mass size with significant functional and symptomatic improvement. 

## Discussion

Testicular germ cell tumors (GCTs) are the most common malignancy in men between the ages of 20 to 39 years [4]. There are many pathological subtypes of testicular GCTs including seminoma, teratoma, embryonal, yolk sac carcinoma, and choriocarcinoma [4]. Seminoma, arising from the seminiferous tubules, is a malignant GCT that most commonly affects the testicle [5]. Other sites of seminoma formation include extra-gonadal sites such as the mediastinum and retroperitoneum. Seminoma is associated with a promising survival rate of 95% if it is discovered and treated early [6,7]. The etiology of seminoma is unknown; however, several factors have been implicated including genetic alteration, infection, environmental exposure, history of cryptorchidism, trauma, maternal estrogen exposure, and family history [8-12].

Seminoma most commonly presents as a painless testicular mass, which is often noticed incidentally by a patient or partner [5]. Metastatic disease may present with symptoms indicative of tumor spread, such as a neck mass, gastrointestinal sequelae, bone pain, or neurological symptoms. Workup of a seminoma includes biopsy and immunohistochemical analysis [13]. Serum tumor markers such as alpha-fetoprotein, LDH, and beta-human chorionic gonadotropin (beta-HCG) are measured to aid in diagnosis. Serum biomarker aberrations seen in some cases of seminoma include elevated beta-HCG, LDH, and placenta-like alkaline phosphatase [14]. Scrotal US is obtained to evaluate for other conditions and typically shows a homogenous hypoechoic mass. In rare cases, there may be cystic and calcified regions, although these findings are more consistent with a non-seminoma. Orchiectomy is the therapy of choice for both diagnosis and treatment [15]. Following diagnosis, evaluation for metastasis is conducted using a combination of x-ray, CT, and MRI. Treatment is then initiated depending on tumor stage, as determined using either International Union Against Cancer (IUAC) or American Joint Commission on Cancer (AJCC).

This case highlights the importance of recognizing DVT as a possible first presenting sign of testicular cancer in non-verbal or cognitively impaired patients, as may be seen in a subset of patients with DS. This is especially important in patients with DS where there exists a higher prevalence of testicular cancer. It further demonstrates the need for providing individualized care to patients who cannot provide a thorough history by supplementing clinical guidelines with clinical judgment. Moreover, considering potential communication issues, we recommend annual surveillance for testicular cancer in patients aged 15-45 years with DS. A similar suggestion was previously made by Rethore et al. for health professionals in Europe [16]. However, there are no consensus testicular cancer screening guidelines in the United States.

## Conclusions

Extensive DVTs may be the initial presenting sign of an occult testicular malignancy. This is especially true in patients with DS where typical presenting features of testicular cancer may be missed. In addition, we recommend annual testicular examination in otherwise asymptomatic patients with DS, especially in patients 15-45 years of age.
